# Polyimine-Based Self-Healing Composites: A Review on Dynamic Covalent Thermosets for Sustainable and High-Performance Applications

**DOI:** 10.3390/polym17121607

**Published:** 2025-06-09

**Authors:** Xiaoxue Wang, Si Zhang, Yun Chen

**Affiliations:** School of Mechanical Engineering, Jiangsu University of Science and Technology, Zhenjiang 212000, China; 15505157601@163.com

**Keywords:** polyimine, composite, dynamic covalent thermoset, self-healing materials, recyclable polymers, engineering application

## Abstract

Polyimine-based composites have emerged as a promising class of dynamic covalent thermosets, combining high mechanical strength, thermal stability, self-healing, recyclability, and reprocessability. This review systematically summarizes recent advances in polyimine synthesis, highlighting dynamic covalent chemistry (DCC) strategies such as imine exchange and reversible Schiff base reactions. Structural customization can be achieved by incorporating reinforcing phases such as carbon nanotubes, graphene, and bio-based fibers. Advanced fabrication methods—including solution casting, hot pressing, and interfacial polymerization—enable precise integration of these components while preserving structural integrity and adaptability. Mechanical performance analysis emphasizes the interplay between dynamic bonds, interfacial engineering, and multiscale design strategies. Polyimine composites exhibit outstanding performance characteristics, including a self-healing efficiency exceeding 90%, a tensile strength reaching 96.2 MPa, and remarkable chemical recyclability. Emerging engineering applications encompass sustainable green materials, flexible electronics, energy storage devices, and flame-retardant systems. Key challenges include balancing multifunctionality, enhancing large-scale processability, and developing low-energy recycling strategies. Future efforts should focus on interfacial optimization and network adaptivity to accelerate the industrial translation of polyimine composites, advancing next-generation sustainable materials.

## 1. Introduction

**Figure 1:**
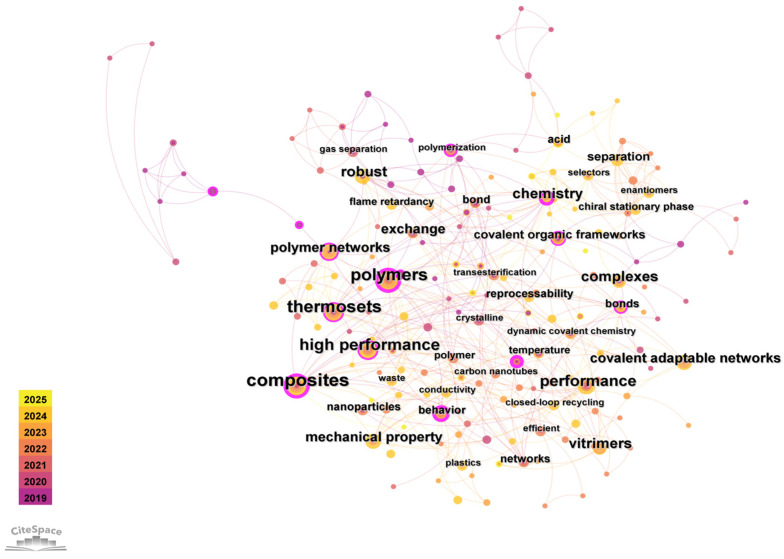
Bibliometric statistics for the past six years.

Polyimine (PI) refers to a family of polymers featuring dynamic imine bonds (C=N), typically generated by condensation reactions between primary amines and aldehydes or ketones [1]. As highlighted by bibliometric analysis (Figure 1), the field’s evolution over the past six years—marked by high-frequency keywords like ‘polymer’, ‘chemistry’, ‘thermoset’, ‘vitrimers’, ‘composites’, ‘covalent adaptable networks (CANs)’, and ‘high performance’—demonstrates a clear trajectory from fundamental chemical exploration towards leveraging dynamic covalent chemistry (DCC) to transform PIs into reprocessable, high-performance composites. The underlying reaction mechanism, known as the Schiff base reaction, involves dehydration–condensation between nucleophilic amines and carbonyl groups, leading to the formation of reversible imine linkages [2,3]. Imine bonds exhibit reversible exchange and cleavage, enabling pH-regulated hydrolysis and reformation in aqueous solutions. These bonds further undergo dissociation or metathesis when catalyzed, as demonstrated in prior studies [4]. Such reversibility allows polyimines to act as dynamic covalent networks (CANs), endowing them with responsiveness to stimuli and the potential for self-healing, recyclability, and reprocessability. Compared to traditional thermosetting polymers such as epoxy resins, PI-based networks not only offer superior thermal and mechanical performance but also enable reversible topological reconfiguration, effectively addressing the irreparable and non-recyclable limitations of conventional thermosets [3].

Recent studies have focused on enhancing the multifunctionality of polyimines by tuning both their molecular design and composite structure. Strategies include the incorporation of organic and inorganic reinforcements, such as carbon nanotubes, graphene, and natural fibers, to significantly improve their mechanical, electrical, and thermal properties. Interfacial engineering, combined with dynamic bond architecture, has been shown to improve stress transfer efficiency within the network, further boosting overall performance. Polyimines have thus become promising candidates for applications in flexible electronics, flame-retardant coatings, and environmentally friendly adhesives. Meanwhile, diverse synthesis techniques such as solution casting, hot pressing, and interfacial polymerization have been developed, offering flexible control over morphology. The integration of bio-based monomers (e.g., lignin and vanillin) into PI systems has further advanced their sustainability, enabling the upcycling of waste into high-performance adhesives and aerogels via closed-loop regeneration processes aligned with circular economy principles.

Building upon previous developments, this review systematically summarizes the synthesis strategies, dynamic bond design, composite fabrication methods, and reinforcement optimization approaches for polyimine-based materials. It highlights the interplay between dynamic covalent chemistry and interfacial engineering in achieving superior mechanical strength, self-healing capability, recyclability, and multifunctionality. By comprehensively analyzing recent advancements across flexible electronics [5], self-healing waterproof coatings [6], and energy storage systems [7], this work identifies critical challenges such as balancing multifunctionality, enhancing large-scale processability, and optimizing low-energy recycling strategies that are driven below 80 °C. Furthermore, this review emphasizes future research directions toward interfacial optimization, dynamic network adaptivity, and sustainable manufacturing pathways, aiming to accelerate the industrial translation of polyimine composites into next-generation high-performance and eco-friendly materials.

## 2. Synthesis of Polyimines via Dynamic Chemistry Approaches

### 2.1. Dynamic Imine Chemistry

**Figure 2:**
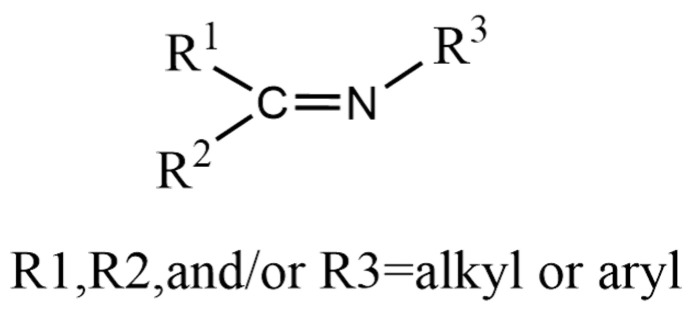
Molecular structure of imines.

From a structural perspective, imines are classified as organic compounds in which the oxygen atom of a carbonyl group (aldehyde or ketone) is substituted by a nitrogen atom [8], typically following the general formula RC=NR’, where R and R’ represent hydrocarbon groups or hydrogen atoms, as illustrated in Figure 2 [9,10]. Imine chemistry, often referred to as Schiff base chemistry, involves two primary processes: the condensation/hydrolysis of imine bonds and dynamic imine exchange [11]. These reversible covalent interactions are widely employed in the fabrication of self-healing materials [12]. To illustrate, Taynton et al. [13] demonstrated that malleable polyimine networks could serve as adhesives for woven carbon fiber composites, facilitating efficient closed-loop recycling by allowing complete recovery of both fibers and adhesives [11]. By disrupting the stoichiometric balance of polyimine networks with excess free primary amines, the adhesive dissolves, achieving 100% recycling of carbon fiber composites without additional chemicals or energy consumption. The reversible nature of imine bonds (C=N) not only imparts thermoplasticity to polyimines but also confers self-healing capabilities, allowing materials to exhibit excellent machinability and repair under heat or moisture stimulation.

**Figure 3:**
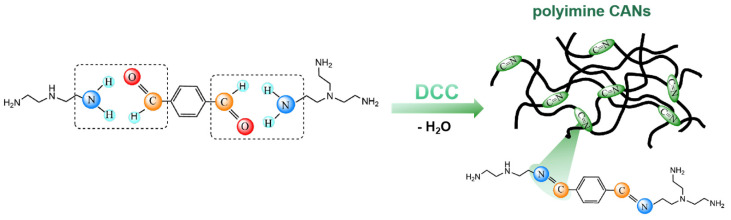
Synthesis of polyimines via dynamic chemistry approaches.

While conventional imine bonds (Schiff bases) formed from ammonia/primary amines and aldehydes/ketones exhibit poor stability and water sensitivity, limiting their application scope, dynamic covalent chemistry (DCC) provides a robust alternative for synthesizing polyimines [4]. The dynamic chemical synthesis method for polyimine is illustrated in Figure 3. Through the integration of alkyne metathesis with imine chemistry, Zhang et al. [14] successfully fabricated conjugated polymers, porous frameworks, and stretchable covalent network polymers. Covalent adaptive networks (CANs), defined as polymers crosslinked via reversible covalent bonds [15], merge the advantages of thermosets (e.g., dimensional stability, mechanical strength) [16] and thermoplastics (reprocessability) [17].

**Figure 4:**
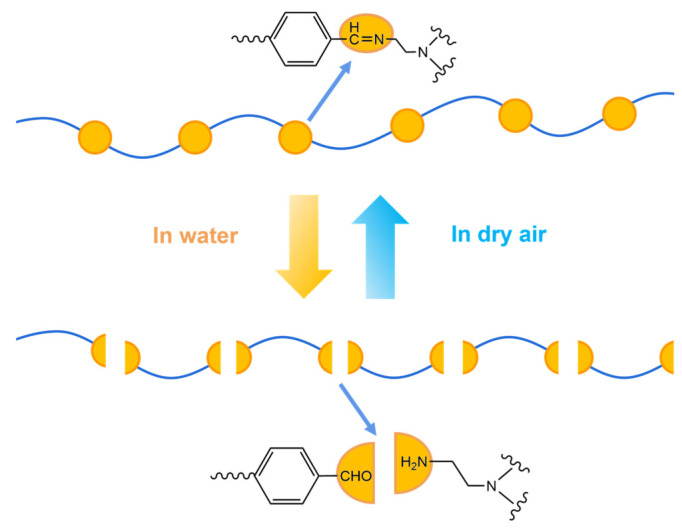
Schematic diagram of water-driven dynamic imine bond degradation.

The incorporation of dynamic covalent bonds has positioned polyimine (PI) as a high-performance smart material. Through reversible bond breaking and reforming, dynamic covalent chemistry enables materials to respond adaptively to environmental triggers, including temperature, pH, and light [12,18]. This property imparts self-healing, shape memory, and other adaptive functions, broadening PI’s application potential. As shown in Figure 4, water can trigger the reversible transition of dynamic covalent bonds. Heat-driven extensibility arises from imine bond exchange reactions, initiated at elevated temperatures (e.g., 80 °C), which relax internal stresses and enhance ductility. Integrating dynamic bonds with bio-based monomers enables the development of sustainable thermosets, reducing reliance on petroleum resources [3]. Taynton et al. [12] reported a catalyst-free, water-driven recyclable polyimine network, achieving green, energy-efficient processing. This material behaves as a thermoset at room temperature and is ideal for load-bearing applications and DIY prototyping. With the continuous in-depth research on the recyclability of polyimines, Wang et al. [19] proposed an innovative ‘aerogel-sol-aerogel (ASA)’ approach to recycle, repair, and reprogram polyimine aerogels. This method decomposes aerogels into oligomer solutions and reconstructs them into new aerogels, offering high repeatability and selectivity for hybrid waste streams.

### 2.2. The Methods for Adapting Polyimine to Different Molding States

The selection of polyimine fabrication strategies is critically governed by target product morphology and functionality requirements, as systematized in Table 1. Solution casting enables uniform dispersion for films, gels, and composites but faces solvent recovery challenges; hot pressing offers scalability for bulk materials and multilayers, yet risks interfacial weakness; interfacial polymerization achieves ultrathin 2D films and functional coatings, though it requires precise reaction control. This methodological versatility allows precise tuning of structural integrity, interfacial engineering, and dynamic adaptability across diverse applications.

#### 2.2.1. Solution Casting

**Figure 5:**
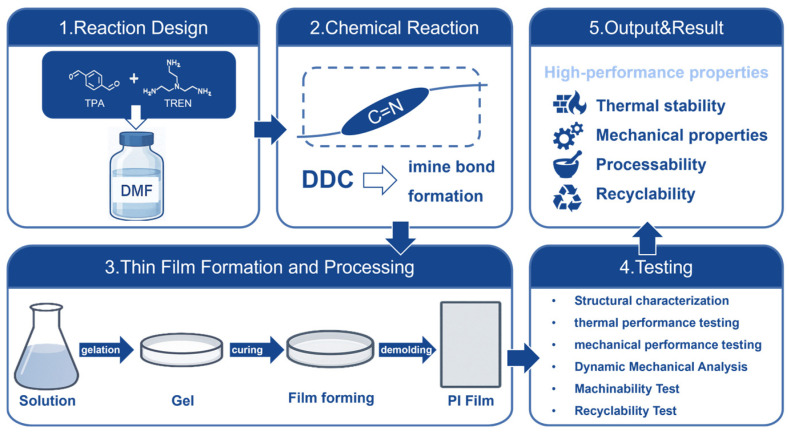
General fabrication flowchart of polyimine films (exemplified by PI-T) [26].

The selection of polyimine fabrication methods requires optimization based on the target product morphology. Solution casting leverages monomer dissolution in organic solvents to prepare polyimine solutions and derived materials. Precise control over solution concentration and reaction parameters enables directional construction of polymer chain architectures. Lei’s team [26] exemplifies this general solution casting process (Figure 5) to produce robust, transparent polyimine films (~0.15 mm thick). They dissolved diamines (ADH or ODA) and triamine (TREN) with TPA in DMF (aldehyde/amine ratio = 1:1), varied the diamine/triamine ratio, then heated (80 °C, 10 min) to induce gelation via Schiff base reactions, followed by vacuum drying.

**Figure 6:**
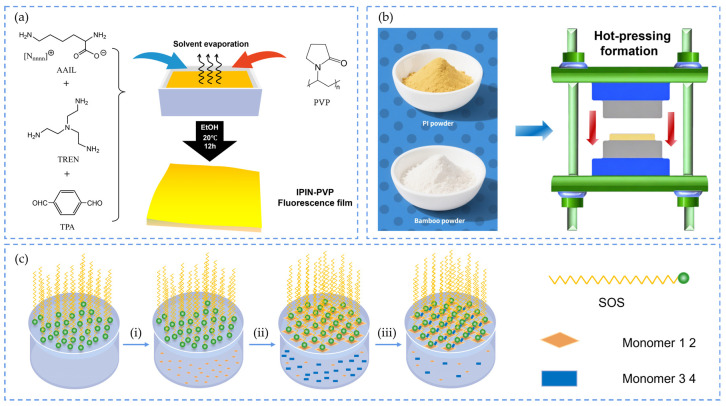
(**a**) Solution casting of IPIN-PVP membranes [27]; (**b**) hot pressing of BP/PI composites [33]; (**c**) SMAIS of PI-2DP films at air/water interfaces: (i) create surfactant monolayer on water surface. (ii) Arrange monomer 1,2 underneath surfactant monolayer. (iii) Carry out polymerization at interface [34].

It is very common to prepare polyimine cross-linked networks by solution casting method. For example, a mixture of lysine-based amino acid ionic liquids (AAILs), tris(2-aminoethyl)amine (TREN), terephthalaldehyde (TPA), and polyvinylpyrrolidone (PVP), was dissolved in ethanol [27]. Schiff base reactions between amino and aldehyde groups produced imine bonds, forming an IPIN-PVP solution, which upon ethanol evaporation, yielded fluorescent composite films [27] (as shown in Figure 6a). For instance, Tian et al. [28] synthesized polyimine thermosets by dissolving monomers in chloroform, followed by solvent evaporation and curing. Similarly, Yu et al. [29] synthesized semi-aromatic polyimine matrices using 3,4′-ODA and TA monomers, with aliphatic TREN as a crosslinker, and formed composite films via solvent evaporation.

#### 2.2.2. Hot Pressing

Hot pressing combines heat and pressure to induce plastic deformation or chemical crosslinking, enabling efficient fabrication of dense polyimine composites. Generally, researchers first grind the prepared solid material into powder, then place it into a hot press for thermal compression (as shown in Figure 6b). Su et al. [30] prepared Cel-PI films by hot pressing moistened Cel-PI powder between polyimide membranes at 70 °C and 30 MPa for 20 min. Zhang et al. [31] achieved homogeneous wood flour/polyimine composites by blending powders via planetary ball milling and hot pressing at 80 °C and 9 MPa. This method ensures smooth surfaces, strong interfacial adhesion, and enhanced mechanical performance. Similarly, Zhang et al. [32] blended varying amounts of wood flour with bio-based polyimine vitrimers to prepare lignocellulose-based composite films. The mixture was hot-pressed at 120 °C and 10 MPa for 5 min [32]. This method produced polyimine composites with smooth, uniform, and defect-free surfaces. Additionally, it ensured strong interfacial compatibility between reinforcements and the matrix, thereby enhancing overall material performance.

#### 2.2.3. Interfacial Polymerization

Interfacial polymerization occurs at immiscible liquid interfaces (e.g., water–oil) to form stable interfacial layers, improving mechanical and thermal properties. Dai et al. [35] pioneered air/water interfacial synthesis of covalent monolayers using dynamic chemistry, opening new pathways for the synthesis of two-dimensional polymers. Zhang et al. [36] fabricated highly crystalline polyimine films by confining protonated TAPP monomers and DhTPA at water surfaces. Ge et al. [37] developed amorphous 3D-PPIn nanofilms via interfacial oligomer splicing, achieving rapid molecular/ion differentiation. This method is also pivotal in water treatment. Baig et al. [38] synthesized thin-film composite (TFC) membranes through interfacial reactions of aromatic diamines and acyl chlorides. Interfacial polymerization also overcomes the challenge of integrating insoluble covalent organic framework (COF) powders into thin-film electronics. By developing the surfactant monolayer-assisted interfacial synthesis (SMAIS) strategy, Sahabudeen et al. [34] realized the fabrication of PI-2DP films at the air/water interface, leading to the first successful synthesis of 2D COF films (as shown in Figure 6c).

## 3. Preparation of Polyimine Composites

### 3.1. Selection of Reinforcements

The choice of reinforcements significantly influences the performance of polyimine composites. Based on their properties, reinforcements can be categorized into organic and inorganic phases. As shown in Table 2, organic reinforcements mainly enhance toughness and processability, while inorganic phases significantly improve strength, conductivity, and thermal stability. Their effects stem from different mechanisms—hydrogen bonding in organics versus interfacial load transfer in inorganics—highlighting the need for application-oriented selection.

#### 3.1.1. Organic Reinforcements

Organic reinforcements, such as polymers and natural fibers, enhance the mechanical properties and processability of composites through their inherent flexibility and toughness. Organic reinforcements play a critical role in polyimine composites. While polyimines inherently exhibit excellent thermal stability and mechanical strength, their processability and toughness remain limited. For example, Zhang et al. [42] synthesized self-healing polyimine networks using peach gum polysaccharide (PGP) and chitosan (CS), achieving a tensile strength of 56.5 MPa and 97.8% healing efficiency. Su et al. [43] filled the pores of cellulose paper with polyimine, functionalized cellulose paper with polyimine covalent adaptive networks (CANs), creating paper–polyimine composites (PPCs) with high mechanical strength (71 MPa), water resistance, and full recyclability. In recent years, supramolecular hydrogen bonding motifs have demonstrated unique advantages in regulating network architectures. Ding et al. [44] synthesized a bio-based polyimine (TMP-IPDA-Si) incorporating acylhydrazide structures using lignin-derived vanillin as a precursor. Molecular dynamics simulations revealed that the introduction of acylhydrazone bonds substantially enhanced intermolecular hydrogen bonding interactions (with a hydrogen bond density of 4 nm^−3^), endowing the material with an exceptional tensile strength of 85.7 MPa and a high glass transition temperature of 201.4 °C. Additionally, E. Lamm et al. [39] developed polyimine-coated cellulose nanofibrils (CNF-imine) via aqueous synthesis, improving interfacial compatibility in polymer matrices.

#### 3.1.2. Inorganic Reinforcements

The incorporation of inorganic reinforcements such as carbon nanotubes (CNTs), graphene, and metal oxides enhances the thermal stability, electrical conductivity, and mechanical strength of the composites in a synergistic manner. Single-walled carbon nanotubes (SWNTs) [45] and silicon nanoparticles (SiNPs) [7] not only optimize the tensile modulus of composites but also impart unique conductive properties. Magnetic NdFeB microparticles enable directional control over magnetic performance [46]. ZrO_2_ increased the tensile strength and toughness of polyimine nanocomposites by 40% and 85%, respectively [47]. Furthermore, Mao et al. [48] developed multifunctional composites by incorporating novel inorganic phases (e.g., TiO_2_@MXene heterostructures and sodium vanadium oxide, NVO) into polyimine matrices, achieving both flame retardancy and electrochemical responsiveness. In practical applications, Yu et al. [18] incorporated carbon fiber sheets into a dynamic covalent fluorinated polyimine matrix, resulting in composites that could be welded and reshaped at 80 °C, with water-driven extensibility and reprocessability. In 2022, Wang et al. [49] fabricated bio-based degradable polyimine vitrimers/carbon fiber (CF) composites. While the mechanical properties of CF-reinforced IPDA vitrimers were marginally lower than those of bisphenol A epoxy (E51), polyimine vitrimers exhibited easy degradability in acidic environments and effectively preserved both the surface morphology and chemical structure of recycled CF, promoting the advancement of eco-friendly CF composites [49]. Despite their advantages (e.g., thermal stability and mechanical strength), poor compatibility between inorganic reinforcements and the matrix may lead to uneven dispersion, compromising final performance. Thus, improving interfacial compatibility is a critical research focus. For instance, Zhang et al. [50] modified silicon carbide whiskers (SiCw) and graphene oxide (GO) with 3-aminopropyltrimethoxysilane (APTMS), achieving superior dispersion and enhanced interfacial interactions. Their experiments also confirmed the significant impact of reinforcement content on mechanical properties.

### 3.2. Composite Methods

The diversity of preparation methods for polyimine composites provides extensive possibilities for optimizing material performance. Common approaches include physical blending, chemical bonding, and lamination, each tailored to specific application scenarios and performance requirements.

#### 3.2.1. Physical Blending

Physical blending involves mixing reinforcements with a polyimine matrix via mechanical or solution-based methods. This method is relatively simple to operate and suitable for large-scale production. Cui et al. [33] prepared BP/PI composites through the physical blending of bamboo powder (BP) with polyimine precursors, followed by hot pressing, subsequent to Schiff base crosslinking. The resulting composites exhibited high tensile strength (45.2 MPa), bending strength (121.9 MPa), and solvent resistance, alongside closed-loop recyclability and biodegradability (see Figure 6b). Zhu et al. [46] further incorporated NdFeB microparticles into polyimine solutions to create magnetic soft robots with full chemical recyclability and room temperature self-healing capabilities. During the mixing process, ultrasonic treatment or mechanical stirring is typically employed to ensure uniform dispersion of the magnetic particles in the polyimine solution. While simple and cost-effective, physical blending often yields weaker interfacial bonding compared to chemical methods.

#### 3.2.2. Chemical Bonding

Chemical bonding enhances interfacial strength through covalent interactions between reinforcements and the matrix, thereby breaking through the limitations of traditional physical compounding. Zhu et al. [51] developed superhydrophobic coatings by spraying fluorinated epoxy monomers and Fe_3_O_4_ SiO_2_-NH_2_ nanoparticles onto polyimine films, where epoxy–amine reactions ensured robust adhesion. Wang et al. [52] synthesized Fe_3_O_4_ TAPB-Tp core–shell nanocomposites via interfacial Schiff base polymerization, achieving strong covalent linkages between magnetic cores and polyimine shells. TAPB-Tp acts as the reinforcing phase chemically bonded to the surface of Fe_3_O_4_, thereby achieving the composite of the substrate and the reinforcing phase. However, the reaction process of this method is relatively complex, requiring precise control of reaction conditions to ensure the quality and performance of the final product.

#### 3.2.3. Lamination

**Figure 7:**
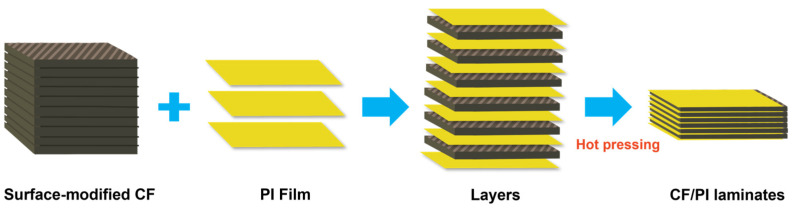
Preparation of CF/PI laminates and schematic representation of interfacial improvement in carbon fiber (CF)/polyimine (PI) laminate [54].

Lamination refers to a composite manufacturing process where distinct material layers, such as fibers, resins, and metals, are stacked using customized techniques to impart integrated properties. Li et al. [53] developed high-performance, recyclable ramie yarn-reinforced polyimine vitrimer composites (RY-PI) by repeatedly hot pressing single-layer RY-PI sheets. Dynamic bond reorganization enabled seamless interlayer integration. Upon completion of polyimine synthesis and carbon fiber (CF) surface modification, Zhang et al. [54] dried the CF fabrics and polyimine (PI) films, then alternately stacked 16 layers of CF fabrics and 17 layers of PI films. The stacked layers were hot-pressed at 100 °C under 5.0 MPa for 10 h, followed by cooling to room temperature, resulting in CF/PI laminated plates (as shown in Figure 7). This approach combines the mechanical advantages of natural fibers (e.g., strength, lightweight) with the reversible nature of dynamically crosslinked polymers, achieving high performance and self-healing capabilities. Innovative chemical/physical recycling strategies further enhance sustainability, positioning lamination as a key technology for replacing traditional plastics and advancing zero-waste goals.

## 4. Factors Affecting the Mechanical Properties of the Composites

The mechanical properties of polyimines and their composites are crucial for engineering applications, prompting extensive research efforts aimed at performance enhancement. A summary of mechanical properties of polyimine materials is provided in Table 3. Numerous studies have explored strategies to optimize network design and reinforcement compatibility. For example, Wang et al. [55] developed fluorinated bio-based polyimine films with a tensile strength of 96.2 MPa, surpassing that of commercial polycarbonate (~65 MPa), demonstrating the potential of tailored polyimine systems in high-performance applications.

### 4.1. Correlation Between Dynamic Bonds and Mechanical Properties

The mechanical behavior of polyimines is predominantly governed by the reversible nature of dynamic imine bonds (C=N). Crosslinking density further modulates mechanical performance. Li et al. [56] demonstrated that increasing crosslinking density in polyimine/epoxy networks enhanced elastic modulus and tensile strength, confirming the critical role of covalent interactions in load-bearing capacity.

**Figure 8:**
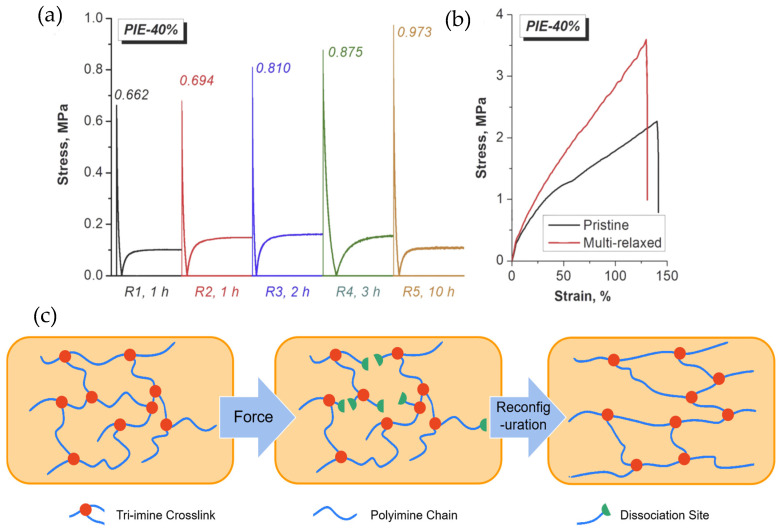
(**a**) Stress relaxation and intensification curves of PIE-40% experiencing successive multiple stress relaxation periods, (**b**) tensile stress–strain curves of pristine and multi-relaxed PIE-40%, and (**c**) schematic illustration of PIE network reconfiguration adapting to deformation [57].

Unlike conventional covalently crosslinked polymers, polyimines enable network rearrangement through dynamic bond exchange, maintaining high mechanical strength while exhibiting stress relaxation and self-healing capabilities. While traditional networks show gradual stress decay under constant strain, polyimine elastomers (PIEs) with dynamic imine bonds can completely relax stress through reversible network reorganization. Notably, Lei et al. [57] observed a unique ‘stress intensification’ phenomenon in PIEs, where stress rebounded after relaxation due to zwitterionic intermediates formed via transamination/transimination reactions (Figure 8a). This contrasts with the monotonic stress decay in conventional networks. The mechanism involves load-bearing chain rupture during deformation, followed by network reorientation through imine exchange (Figure 8c). After multiple relaxation-strengthening cycles, tensile strength significantly increased (Figure 8b), demonstrating how dynamic bonds enable topology optimization for reconfigurable polymer processing.

### 4.2. Interfacial Engineering and Multiscale Performance Optimization

Interfacial interactions between reinforcements and the matrix play a pivotal role in the mechanical optimization of polyimine composites. Zhang et al. [41] incorporated graphene into polyimine matrices, achieving composites with high thermal conductivity and electrical conductivity. Graphene nanoplatelets (GNPs) acted as bridges across microcracks and altered crack propagation paths, thereby enhancing fracture energy dissipation. While covalent bonding provides superior stress transfer efficiency compared to physical adsorption, non-covalent strategies—particularly hydrogen bonding and π-π stacking—offer synergistic enhancements. A representative example is the hydrogen-bonded polyimine thermoset (PI-A/T) system [26], where amide-containing ADH monomers introduce reversible hydrogen bonding sites. This dual-network design achieves an optimal balance: hydrogen bonds restrict chain mobility to enhance stiffness, while their dynamic break/reformation dissipates energy under stress, preserving toughness. Further interfacial reinforcement is demonstrated in Lyu’s vanillin-based graphene/polyimine composites [41]. Here, π-π stacking between aromatic rings (abundant in both the polyimine matrix and graphene nanosheets/GnPs) reduces free volume and promotes dense chain packing. The GnPs not only strengthen this stacking network but also suppress phonon scattering, leading to concurrent improvements in mechanical strength and thermal conductivity. Such multi-mechanism interfacial engineering exemplifies how dynamic covalent networks can integrate covalent and non-covalent interactions for tailored performance.

**Figure 9:**
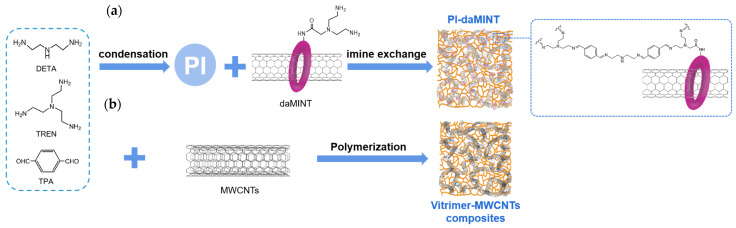
(**a**) Schematic diagram of PI-daMINT chemical ligation composite method [45]; (**b**) schematic diagram of physical blending method for Vitrimer-MWCNTs [40].

As illustrated in Figure 9, the two composite systems share the same polyimine matrix but differ significantly in how carbon nanotubes are incorporated. In the PI-daMINT system (Figure 9a), a specially designed U-shaped macrocycle acts as a molecular bridge that connects single-walled carbon nanotubes (SWNTs) with the polyimine network. The macrocycle wraps around SWNTs through non-covalent interactions, while its terminal groups enable chemical bonding with the imine matrix. After thermal treatment, the resulting daMINTs integrate covalently into the network, ensuring strong interfacial adhesion [45]. In contrast, the Vitrimer-MWCNT composite (Figure 9b) is formed via physical blending without chemical linkage. Although good dispersion is achieved, the lack of interfacial bonding limits mechanical performance and leads to delamination under repeated stress [40]. This comparison highlights the critical role of covalent integration in enhancing the reinforcement efficiency of nanofillers.

### 4.3. Impact of Self-Healing Behavior on Mechanical Performance

**Figure 10:**
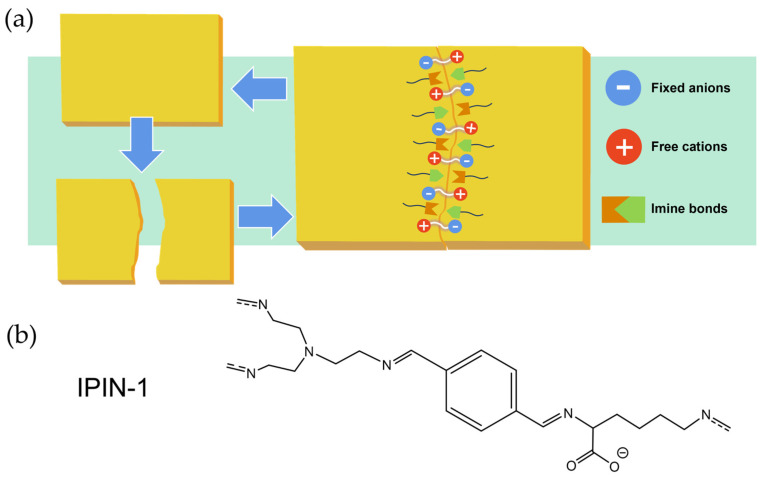
(**a**) Schematic diagram of self-healing principle of IPIN-1. (**b**) Molecular formula of IPIN-1 [58].

The self-healing behavior of polyimines, driven by the reversible reorganization of dynamic bonds (e.g., transamination of imine bonds or ionic interactions), significantly restores mechanical properties. For example, Peach Gum—Chitosan based PI networks (PGCS) retained 97.3% strength after two physical reprocessing cycles, while the ionic polyimine network (IPIN) maintained mechanical performance comparable to virgin materials after acidolysis-repolymerization closed-loop recycling [42,58]. According to experimental findings, IPIN-1 achieved a tensile strength of 4.13 MPa and an elongation of 180% after 1 h of self-healing, reaching 4.21 MPa and 241% after 12 h, with a recovery rate over 96%. Cyclic tensile tests showed a low hysteresis and 19.8% residual strain after five cycles, indicating good mechanical stability. During the preparation of IPIN-1, choline lysinate ([Ch] [Lys]) was used, where the hydroxyl group of [Ch]^+^ participated in hydrogen bonding with iodine. The presence of hydrogen bonds enhances intermolecular interactions, thereby improving the stability and recovery capability of the material during cycling. Additionally, the dynamic reorganization of imine bonds, coupled with electrostatic interactions, constitutes the predominant mechanism responsible for the robust self-healing capability inherent to the material (as shown in Figure 10). The healed material could lift 1000 g, demonstrating excellent load-bearing capacity.

However, current systems face limitations such as toughness reduction after multiple repairs. In the experiments conducted by Yang et al. [59], compared to the original elastomer, after the first, second, and third reprocessing cycles, the tensile strength recovered to 96.0%, 86.5%, and 80.6% of the original values, respectively; the elongation at break reached 96.2%, 89.8%, and 79.3% of the original values, respectively. They systematically studied the self-healing behavior at room temperature. The tensile strength recovered to 0.75 MPa after 6 h, 1.74 MPa after 12 h, and 2.41 MPa after 24 h, corresponding to recovery efficiencies of 30.0%, 69.3%, and 96.0%, respectively. Increasing the healing temperature to 40 °C and 60 °C improved 6 h efficiencies to 61.0% and 84.9%, respectively, confirming the thermal acceleration of dynamic bond reorganization [59]. Future strategies should focus on room temperature triggers (e.g., light/moisture response) and in situ repair technologies for complex components.

**Table 3:** Mechanical performance of polyimine materials (20 to 25 years).

Polyimine Materials	Physical Form	Mechanical Property Analysis						Key Findings	Ref.
		Tensile Strength (MPa)	Elongation at Break (%)	Young’s Modulus (GPa)	Thermal Stability (Td5%, °C)	Self-Healing Efficiency (%)	Limiting Oxygen Index (LOI, %)		
IPIN	film	4.28	250	-	>160	Self-healing efficiency: 50.04% after 5 min	-	Exhibits good ductility, self-healing ability, and recyclability. Electrochemical sensors fabricated from IPIN-1 demonstrate high response rates and low detection limits for iodine monitoring.	[58]
PE0.5-0.2 vitrimer	film	2.51	1158	-	-	Self-healing at room temperature: 30.0 (6 h), 69.3 (12 h), 96.0 (24 h)	-	The material was synthesized through a simple two-step one-pot process at room temperature, exhibiting outstanding mechanical properties, self-healing capability, degradability, and reprocessability.	[59]
CTM-3	film	0.56 ± 0.66	38.4 ± 0.3	-	455.0	-	41.6	A catalyst-free vitrimer featuring enhanced flame retardancy, thermal stability, solvent resistance, mechanical strength, and recyclability, outperforming conventional counterparts in reported studies.	[60]
PIM-4	film	94.5 ± 2.6	6.1 ± 0.9	3.5 ± 0.2	434	-	-	Exhibits low water uptake (~0.14–0.15%), excellent mechanical properties minimally affected by absorbed water, chemical resistance, and recyclability, positioning it as a promising alternative to petroleum-based thermosetting resins for harsh environments.	[61]
FA-100	film	28.47 ± 2.01	7.74 ± 1.24	0.37	161.32	-	28.8	Elevating D-FA content enhances the network’s crosslink density and mechanical properties. Dynamic imine linkages endow the material with reprocessability and acid-degradability. FA-100 demonstrates superior flame resistance, achieving a limiting oxygen index (LOI) of 28.8%.	[62]
CO-PIM-75	film	62.5	12.9	-	>242	-	-	High thermal stability. Tensile properties are improved by adjusting the 2,4-ODA/6FAPB ratio, achieving performance comparable to PC. Exhibits good hydrolytic and solvent resistance, with negligible deterioration in mechanical properties after recycling.	[63]
Cel-PI	film	46.3	2.2	2.9	-	-	-	Characterized by dynamic network exchange and an amorphous structure, the material achieves excellent thermal processability, mechanical robustness, water/solvent resistance, thermal stability, and recyclability.	[30]
PGCS-100	film	56.5	20.6	0.439	227.1	Up to 97.8%	56.5	Demonstrates outstanding mechanical properties, high thermal stability, self-healing, welding ability, shape memory, reprocessability, and chemical recyclability.	[42]
PI-A1.0/T1.0	film	55.2	72	1.7	-	-	-	Incorporating hydrogen bond crosslinking significantly enhances mechanical strength and stiffness without sacrificing ductility, offering an effective strategy for strengthening and toughening dynamic covalent thermosets.	[26]
2D Polyimine Films	film	-	6.5 ± 2.4	8.6 ± 2.5	-	-	-	In situ TEM tensile testing allows nanoscale observation of structural evolution and fracture dynamics. Crack initiation preferentially occurs along (100)/(010) directions, with chemical structure influencing mechanical failure.	[64]
CO-PIM-75	film	62.5	12.9	-	>242	-	-	High thermal stability. Tensile properties are improved by adjusting the 2,4-ODA/6FAPB ratio, achieving performance comparable to PC. Exhibits good hydrolytic and solvent resistance, with negligible deterioration in mechanical properties after recycling.	[63]
Cel-PI	film	46.3	2.2	2.9	-	-	-	Characterized by dynamic network exchange and an amorphous structure, the material achieves excellent thermal processability, mechanical robustness, water/solvent resistance, thermal stability, and recyclability.	[30]
PGCS-100	film	56.5	20.6	0.439	227.1	Up to 97.8%	56.5	Demonstrates outstanding mechanical properties, high thermal stability, self-healing, welding ability, shape memory, reprocessability, and chemical recyclability.	[42]
PI-A1.0/T1.0	film	55.2	72	1.7	-	-	-	Incorporating hydrogen bond crosslinking significantly enhances mechanical strength and stiffness without sacrificing ductility, offering an effective strategy for strengthening and toughening dynamic covalent thermosets.	[26]

## 5. Engineering Applications of Polyimine and Composites

**Figure 11:**
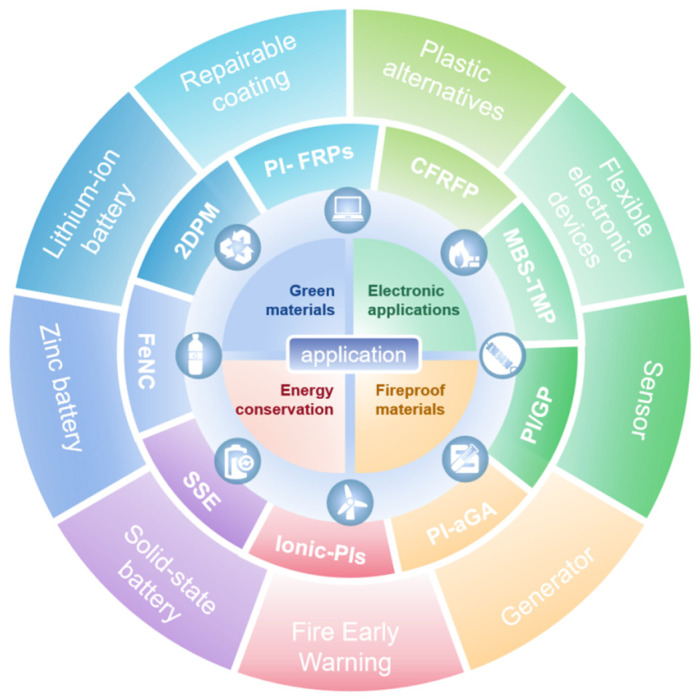
Engineering applications of polyimines and composites.

Polyimines and their composites exhibit transformative potential across diverse engineering fields, driven by their unique combination of dynamic covalent adaptability, mechanical robustness, and multifunctional integration. Figure 11 illustrates the various applications of polyimine and its composite materials. Meanwhile, in Table 4, we summarize the applications of polyimines and their composite materials in engineering over the period of 20 to 25 years.

### 5.1. Green Materials

**Figure 12:**
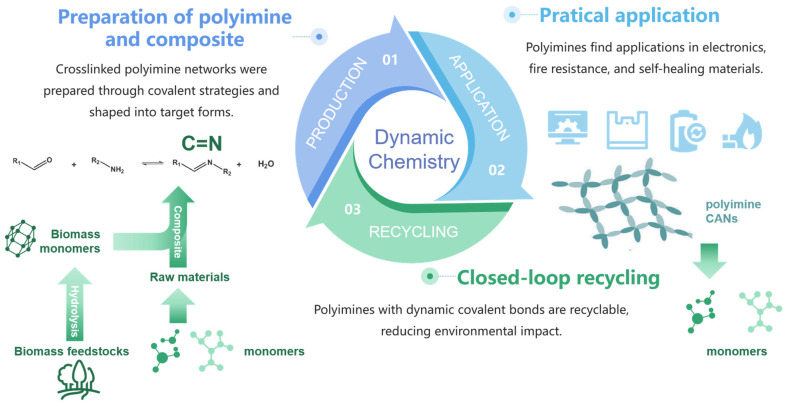
Closed-loop recyclable polyimines and composites enabled by dynamic covalent chemistry.

As global industrialization accelerates, the demand for sustainable alternatives to traditional petroleum-based thermosets has intensified [79]. Therefore, the development of high-performance, recyclable, and biodegradable green materials has become an important research direction in the field of materials science. Polyimines address this need through closed-loop recyclability, biodegradability, and bio-based monomer utilization [80]. Biomass-reinforced recyclable polyimine composites exemplify green materials through dynamic imine chemistry; Figure 12 illustrates this recycling process. It has obvious advantages compared with other traditional materials. For example, although traditional polyimide materials possess excellent thermal and mechanical properties, their high coefficient of friction and wear rate significantly impact service life. Polyimines, through the design of dynamic covalent bonds, exhibit excellent recyclability. Moreover, the introduction of modified fillers and reinforcing phases can significantly enhance their tribological properties.

In the field of green materials, polyimine composites have driven significant advancements in sustainability through innovative applications. Su et al. [81] incorporated imines and amines into polymer networks, establishing an efficient approach for converting biomass into eco-friendly plastics characterized by excellent repairability, renewability, and closed-loop recyclability. These materials accommodate up to 70% wood-derived biomass and display mechanical properties comparable to or exceeding those of traditional plastics [81]. Xiong et al. [21] upcycled non-recyclable polystyrene (PS) into high-performance poly(styrene-imine) aerogels (PSAs) via post-functionalized amination and Schiff base reactions. These aerogels undergo quantitative depolymerization under acidic conditions, enabling infinite material regeneration. Jia et al. [82] developed high-performance bio-based polyimine materials (Bio-Si-PABZs) using a catalyst-free copolymerization strategy. By integrating rigid and flexible structures with dynamic covalent bonds, supramolecular interactions, and hydrogen bonding, these materials demonstrated exceptional properties, including high strain tolerance, tensile strength, thermal stability, self-extinguishing behavior, self-healing capability, and recyclability. Notably, waste from these materials could be repurposed into high-performance adhesives. Li et al. [83] further engineered a series of high-performance polyimine composites by incorporating bio-based monomers and degradable crosslinkers, achieving closed-loop recyclability and biodegradability. Additionally, bio-based resources such as vanillin, bamboo powder, starch, chitosan, and natural peach gum have been widely utilized for polyimine functionalization, further expanding their eco-friendly applications.

Polyimine composites demonstrate outstanding mechanical strength, self-healing, anti-creep, and solvent resistance in coatings. Reprocessability enables heat/pressure-driven restoration of damaged coatings, while self-healing autonomously repairs microcracks. Their dimensional stability under prolonged stress and chemical durability further enhances applicability. To optimize transparency and waterproofing, polydimethylsiloxane (PDMS) has been integrated into polyimine matrices, yielding materials with dense crosslinking, optical clarity, water resistance, UV protection, and self-healing. Xie et al. [6] developed self-healing waterproof coatings by condensing fractionated lignin with PDMS and oleylamine, achieving high crosslinking density and near-invisible post-repair scratches. Similarly, Wang et al. [84] synthesized hyper-crosslinked HHMOP membranes with 91.35% healing efficiency at 25 °C/60% RH within 24 h, showcasing promise for advanced coatings.

### 5.2. Electronic Applications

In addition to their outstanding green material properties, polyimine-based composites exhibit unique advantages in functional electronics. Their combination of electrical performance and mechanical flexibility offers new pathways for next-generation devices. By doping nanoscale conductive fillers and optimizing structure, PI composites have been developed for wearable systems, energy storage, and intelligent sensors. Their intrinsic network reconstruction and closed-loop recyclability also provide sustainable solutions for electronic waste management, aligning device lifecycles with circular economy principles.

The flexible electronics breakthroughs of PI composites stem from their structure–function synergy. This material system successfully achieves the unification of mechanical deformation adaptability and functional stability through the synergistic effect of dynamic network design and nano-conductive phases. For example, MWCNT-reinforced PI networks enable programmable reconfiguration and electrical percolation, ideal for durable wearables. He et al. [67] fabricated reconfigurable 3D sensors from PI/MWCNT inks, achieving electrothermal-driven self-healing. It is particularly noteworthy that this system exhibits electrothermal response-driven interfacial bond reconfiguration characteristics during the damage repair process. This intelligent repair mode provides an innovative solution for the full lifecycle management of electronic devices. Zhang et al. [5] fabricated monolithic PI/graphene aerogels exhibiting a compressive strength of up to 1.2 MPa, electrical conductivity of 79 S/m, and excellent durability after 3000 compression cycles, emphasizing their potential for sustainable sensing applications.

**Figure 13:**
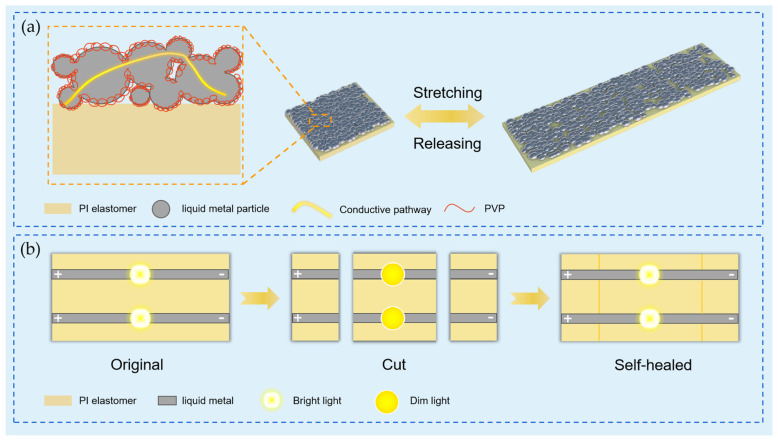
(**a**) A schematic illustration of the sensing mechanism of the sensor; (**b**) a schematic diagram of the self-healing process of the conductive pathways of the flexible strain sensor [59].

Polyimine composites have achieved notable advancements in sensor technology. Yang et al. [59] developed room temperature polyimine elastomers with mechanical robustness, self-healing, and recyclability for liquid metal (LM)-based strain sensors. The sensors were fabricated by dip-coating elastomer strips with PVP-coated liquid metal (LM) conductive ink and evaporating ethanol. They exhibited crack-free conductivity under 500% strain, stable electrical responses, and repeatable sensing across strains (Figure 13a). After complete severance, the sensors self-healed at room temperature within 24 h (Figure 13b). The elastomer substrate fully degraded in 0.1 mol/L HCl/DMAc (600 min), enabling LM recovery via centrifugation for closed-loop recycling. In flexible photodetectors, the integration of tellurium nanowires (Te NWs) and MoS_2_ into polyimine matrices enhanced photoresponse efficiency. Peng et al. [65] fabricated a Te NWs/MoS_2_/polyimine composite photodetector, achieving a rapid response time of 5 s and a specific detectivity of 1.145 × 10^10^ Jones under 532 nm illumination, surpassing the performance of conventional counterparts. Additionally, Qi’s team [85] designed high-performance electrochemiluminescence (ECL) sensors by bridging iridium(III)-polyimine complexes (Ir2PD) with Hf-MOFs, achieving enhanced stability and ECL emission through in situ self-assembly on ITO surfaces.

**Figure 14:**
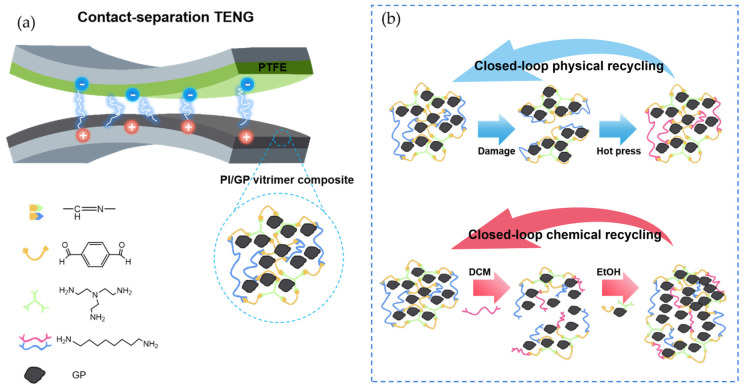
(**a**) Schematic illustration of PI/GP TENG device. Schematic diagram of PI/GP vitrimer composite; (**b**) schematic diagram of physical recycling strategy for PI/GP vitrimer composite. Schematic illustration of the chemical recycling process for the PI/GP [66].

Polyimine composites exhibit remarkable output power and self-healing capabilities in high-performance energy harvesters. Zhu et al. [86] employed dynamic covalent thermosetting PI as a matrix for recyclable hotspot thermoelectric generators (TEGs), achieving post-recovery performance comparable to the original, with excellent mechanical stability under cyclic loading. Figure 14 illustrates the structure and recyclability of PI/GP-based triboelectric nanogenerators (TENGs). In Figure 14a, the composite architecture is shown, where the polyimine matrix, embedded with graphite–polypropylene (GP) powders, enhances surface roughness and dielectric properties [87,88], resulting in high output voltage during mechanical stimulation. Rajabi-Abhari et al. [66] enhanced TENG performance using such PI/GP composites, reaching a power density of 2571 mW/m^2^ under 15 N force at 6 Hz. Figure 14b demonstrates the dual recycling strategies: physically, the composite can be reprocessed via grinding and hot pressing; chemically, it can be depolymerized in a DCM/diamine solution and reconstituted with fresh components. These closed-loop approaches, enabled by dynamic covalent networks, highlight the material’s excellent reusability and sustainability, making it suitable for next-generation wearable and recyclable energy harvesting devices. Additionally, Zhu et al. [86] used PI to encapsulate 200 pairs of Bi_2_Te_3_ and Sb_2_Te_3_ thermoelectric legs with liquid metal electrodes in recyclable, self-healing, and stretchable TEGs (RHS-TEGs). Upon damage, the device could be fully depolymerized in a methanol-based solution, enabling easy separation and reuse of components.

### 5.3. Energy Storage

Polyimine composites offer significant benefits across a range of energy storage technologies, such as lithium-ion batteries, zinc-based batteries, lithium-metal batteries, and metal sulfide/carbon composite systems. Acting as a binder, PI significantly enhances the cycling stability and specific capacity of silicon anodes; as a separator material, it effectively suppresses lithium dendrite growth, thereby improving battery safety and lifespan. Moreover, PI composites achieve high nitrogen doping and three-dimensional superstructures through simple synthetic methods, further enhancing their energy storage performance.

Researchers have utilized the incorporation of nano-silicon into PI matrices as a promising approach to improve the electrochemical performance of silicon anodes. Gao et al. [7] developed a high energy density electrode system by synergistically constructing PI matrices with nano-silicon, using an in situ polymerization approach to form a three-dimensional network. This structure mitigates the volume expansion of silicon, maintaining 80.4% capacity retention after 200 cycles at 95% silicon content, demonstrating excellent structural stability. This interfacial engineering approach offers a novel pathway for advancing the development of high energy density electrodes.

**Figure 15:**
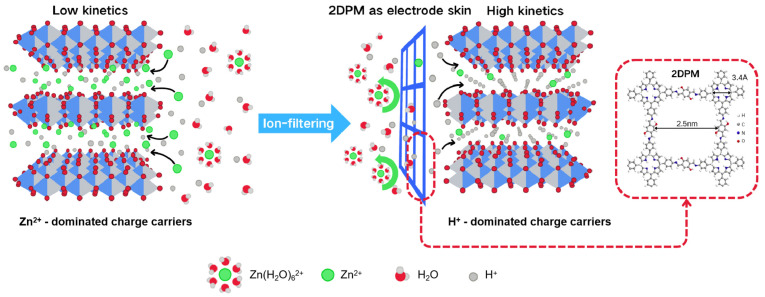
Schematic illustration showing H^+^-dominated cathode intercalation chemistry enabled by H^+^-selective 2DPM coating [89].

In zinc battery technology, two-dimensional polyimine (2DPM) membranes serve as key interfacial coatings due to their proton-selective transport properties. With dual ion-transport nanochannels and abundant proton-conduction sites, 2DPM promotes selective proton transport and accelerates electrode reaction kinetics. By coating a NaV_3_O_8_·1.5H_2_O cathode (active material load 10 mg/cm^2^) with an 80 nm thick 2DPM membrane, Guo et al. [89] reported an areal capacity of 4.5 mAh/cm^2^ and an energy density of 33.8 Wh/m^2^. As shown in Figure 15, the 2DPM membrane enables H^+^-dominated intercalation by creating a highly selective interface that effectively suppresses Zn^2+^ crossover while facilitating fast and directional proton transport. This improves electrochemical kinetics and enhances utilization of the active material, especially in high-mass-loading electrodes. The optimized 2DPM membrane achieved a proton flux higher than 0.9 mol m^−2^ h^−1^ and a H^+^/Zn^2+^ selectivity ratio of 140.7, markedly improving the cathode/electrolyte interfacial properties.

**Figure 16:**
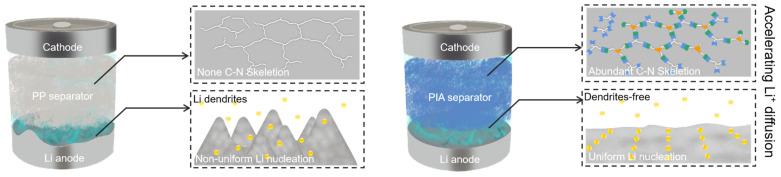
The interfacial Li^+^ diffusion and Li nucleation on the Li metal in PP and PIA separator systems [90].

In lithium-metal batteries, PI aerogels with hierarchical pore architectures and polar chemical environments demonstrate strong dendrite suppression effects. Ding et al. [90] reported that using PI aerogels as functional separators effectively guides uniform lithium deposition via pore structure dispersion and surface functional group interactions. As illustrated in Figure 16, the PI aerogel (PIA) separator enables more homogeneous Li^+^ diffusion and uniform lithium nucleation compared to conventional polypropylene (PP) separators. This helps mitigate dendrite formation and leads to more stable cycling performance. The schematic comparison highlights the critical role of separator chemistry and morphology in regulating interfacial lithium behavior. Batteries utilizing these separators achieved over twice the Coulombic efficiency compared to conventional polypropylene (PP) separators and maintained dendrite-free morphologies during cycling, offering a promising strategy for safe lithium-metal batteries.

As electrode precursors, PI-based materials provide unique advantages for synthesizing metal sulfide/carbon composites. Their molecular design flexibility enables high nitrogen doping, 3D porous architectures, and uniformly distributed nano-sized metal sulfide phases through simple pyrolysis, significantly simplifying the process route. Chen et al. [91] used a flower-like PI superstructure coordinated with metal ions to prepare composites exhibiting superior sodium storage performance. As a representative case, Co_9_S_8_/carbon composites exhibited a reversible capacity of 302 mAh g^−1^ after 200 cycles at 0.5 A g^−1^, demonstrating the validity of this strategy [92].

In the field of solid-state batteries, PI incorporation into solid-state electrolytes (SSEs) can effectively inhibit lithium dendrite formation, significantly extending Li/SSE/Li cell lifespans. However, some drawbacks have been identified. Jiang et al. [93] observed that PI particle distribution within LPS matrices can partially block ion transport pathways, reducing ionic conductivity. Smaller PI particles, while more dispersive, exacerbate this issue. Additionally, PI migration between particles over cycling further decreases conductivity.

### 5.4. Fireproof Materials

Traditional thermosetting materials, such as epoxy and phenolic resins, face significant challenges due to their irrecyclability, limited mechanical performance, poor thermal stability, insufficient fire resistance, environmental incompatibility, and limited dynamic capabilities. These materials typically cannot be reprocessed or recycled through heating, leading to substantial waste generation, resource inefficiency, and environmental pollution [94]. Moreover, traditional thermosets often degrade under high temperature, humidity, or chemically corrosive environments, and combust readily during fires, producing considerable smoke and toxic gases, thereby exacerbating hazards. As summarized in Table 5, traditional thermosets such as epoxy, phenolic, and BMI exhibit high tensile strength and thermal stability but lack recyclability, which limits their long-term sustainability. In contrast, polyimine-based dynamic thermosets combine competitive mechanical and thermal performance with the unique advantage of closed-loop recyclability, positioning them as promising candidates for next-generation green engineering materials.

In contrast, polyimine composites exhibit superior structural integrity at elevated temperatures and leverage reversible dynamic covalent bonds to enable self-healing and recyclability, significantly extending material lifespan and reducing environmental impact. Toldy et al. [76] revealed that polyimine vitrimer systems exhibit superior thermal stability relative to epoxy resins. Flame retardants exhibit more significant reduction in pHRR, with vitreous composites demonstrating superior performance in relative pHRR reduction compared to epoxy composites (as shown in Table 6). Degradation in thermosetting resins is often facilitated by integrating dynamic covalent bonds. Zhang et al. [95] developed a catalyst-free recyclable thermosetting resin (AFD/DCNSA) integrating dual dynamic covalent bonds: pH-sensitive disulfide bonds (-S-S-), which undergo reductive homolytic cleavage to thiyl radicals followed by thiol-disulfide exchange (accelerated at pH > 7), and acetal-type carbon-nitrogen bonds (-C-N-) in hexahydrotriazine rings that degrade via acid-catalyzed reversal of aldoamine condensation. This dual-bond synergy enables three distinct room temperature degradation pathways (acid-triggered, reduction-triggered, or hybrid) while maintaining robust thermal stability (Td5 = 259 °C) and solvent resistance, despite a marginal Tg reduction (116 °C vs. 121 °C for single-bond DDM/DCNSA), overcoming traditional trade-offs between degradability and performance in thermosets. Moreover, Tian et al. [28] designed a polyimine gel (PIT) that can be recycled in acidic solutions and exhibits excellent processability. During combustion, the Schiff base structure undergoes trimerization reactions, forming stable nitrogen-containing hexagonal rings that enhance char layer stability and further improve flame retardancy.

In the selection of reinforcement phases, researchers have gradually shifted from traditional inorganic fillers (such as aluminum hydroxide, magnesium hydroxide, etc.) to nanomaterials (such as MXene, TiO_2_ nanorods, phosphorus-based aromatic compounds, etc.), which exhibit higher flame-retardant efficiency and synergistic effects. Modern fireproof materials not only need excellent fire resistance but also need to integrate other functionalities, such as self-healing and smart responsiveness. For instance, Mao et al. [48] fabricated a TiO_2_@MXene/P, N-containing polyimine nanocomposite (TMPNP) featuring self-healing ability and excellent flame retardancy. By enhancing the composite’s flame-retardant properties and increasing its sensitivity to temperature variations, the TiO_2_@MXene heterostructure demonstrates great potential for application in smart fire warning systems. Additionally, in 2021, Liu et al. [72] developed a polyimine thermoset derived from vanillin-terminated phosphorus-based aromatic monomers (HVP) for fabricating carbon fiber-reinforced composites (CFRPs). Through the introduction of vanillin-terminated phosphorus-based aromatic monomers, Liu et al. achieved simultaneous improvements in flame retardancy and recyclability of CFRPs. Experimental evaluation revealed that the HVP/D230 composite self-extinguished within 1 s after ignition and resisted re-ignition, attributed to the formation of internally combustible carbon layer. Throughout the testing process, no melt dripping was detected. Scanning electron microscopy (SEM) analysis of the residual char morphologies for HVP/D230 and E51/D230 composites indicated that HVP/D230 produced a continuous and uniform surface with distinct stretching tracks, suggesting the formation of a robust, expanded char layer [72]. This well-organized three-dimensional char structure effectively limits combustible gas release, reduces thermal transfer, and significantly enhances the material’s flame-retardant performance.

Researchers have not only improved the flame retardancy of composites but also their mechanical performance, thermal stability, and processability. For instance, Wang et al. (2024) [55] incorporated trifluoromethyl groups into polyimine networks, resulting in a fluorinated bio-based polyimine exhibiting outstanding mechanical strength (tensile strength up to 96.2 MPa), superior flame retardancy (UL-94 V0 rating, LOI of 50%), and excellent reprocessing and solvent-healing capabilities at 160 °C. Yuan et al. (2023) [61] synthesized aromatic polyimine covalent adaptable networks (CANs), significantly enhancing water stability, thermal stability (Td5%: 434–441 °C, Tg: 217–239 °C), and mechanical performance through conjugation effects and aromatic structural stability. These aromatic CANs exhibited very low water absorption (0.14–0.15%) and retained mechanical robustness despite environmental moisture.

### 5.5. Other Applications

#### 5.5.1. Flexible Magnetic Soft Robotics Based on Polyimine Composites

Polyimine (PI) composites exhibit excellent flexibility, stretchability, and responsiveness, offering significant potential in flexible magnetic soft robotics. To enhance recyclability and self-healing, magnetic particles (e.g., NdFeB) were incorporated into PI matrices. In 2023, Zhu et al. [46] developed a dynamic PI network, achieving deformation and locomotion within 2–3 s by adjusting crosslinking density and hydrogen bonding. The robots demonstrated complete chemical recyclability, room temperature self-healing, and retention of mechanical performance after multiple cycles. Similarly, Li et al. [96] reported a mild approach to fabricate magnetic DVA–ETTA–PI nanospheres. The resulting Fe_3_O_4_@PEI@PI nanospheres exhibited fast magnetic response, enriched C-peptides with a detection limit of 0.1 fmol/μL, and maintained performance after at least six reuse cycles, showing potential in chemical sensing and clinical diagnostics.

#### 5.5.2. Applications of Polyimine Composites in Drug Delivery

PI composites, with hierarchical pores and tunable surface chemistry, offer superior drug loading and release properties. Nazanin Mokhtari et al. [97] developed a polyimine-based covalent triazine framework (PI-CTF) to serve as a carrier for sorafenib, attaining an encapsulation efficiency of 98% and a drug loading capacity of 83%. In vitro studies showed pH-dependent release, exhibiting accelerated drug release behavior under acidic conditions (pH 5.3). Cytotoxicity tests confirmed good biocompatibility, indicating PI-CTF’s suitability for targeted drug delivery [97].

#### 5.5.3. Rapid Detection of Volatile Iodine Using Polyimine Composites

Polyimine-based composites show rapid response and high sensitivity in volatile iodine detection. Zhang’s group [27] developed a PI/PVP composite membrane combining the iodine-binding ability of PVP with PI’s selectivity. The sensor responded to trace iodine within 5 s, reducing fluorescence intensity to 62.51% of the initial value, with a detection limit of 4.087 × 10^−8^ mol/L. It maintained excellent selectivity under interference from gases like ethanol and acetone, supporting applications in nuclear monitoring and medical disinfection.

## 6. Conclusions

Polyimine-based dynamic networks, featuring reversible imine linkages (C=N), have significantly mitigated the repairability and reprocessability challenges inherent in traditional thermosetting systems. They have emerged as a critical platform for advancing high-performance and sustainable engineering materials.

This review systematically summarizes the fundamental chemical structures, synthesis mechanisms, dynamic bond design strategies, fabrication techniques, and reinforcement approaches of PI-based composites. Their outstanding performances in mechanical strength, thermal stability, self-healing, electrical responsiveness, and recyclability were comprehensively discussed. Diverse processing methods, including solution casting, hot pressing, and interfacial polymerization, offer versatile pathways for morphology construction and structural tuning of PI systems. The incorporation of various organic and inorganic reinforcements, such as carbon nanotubes, graphene, natural fibers, and bio-based fillers, significantly enhanced the rigidity, toughness, thermal conductivity, flame retardancy, and multifunctionality of PI composites.

Mechanical testing revealed that many PI-based composites achieved tensile strengths exceeding 60 MPa and self-healing efficiencies greater than 90%, outperforming conventional thermosets. The introduction of dynamic covalent networks imparted excellent ductility, stress relaxation behavior, and enhanced interfacial stress transfer and crack suppression capabilities, enabling broad applicability in flexible electronics, wearable devices, high-performance sensors, self-healing waterproof coatings, and green adhesives.

Overall, PI composites, with their tunable chemistry, adjustable architecture, and reconfigurable performance, represent a new paradigm bridging thermosetting and thermoplastic materials, demonstrating great potential for sustainable development and intelligent material design. Nevertheless, further research is needed to address critical challenges, including interfacial coupling stability, functional integration complexity, and scalability toward industrial applications. PI composites are currently at a pivotal stage, transitioning from fundamental research to engineering practice, with significant theoretical and practical implications for future materials science and technology.

## 7. Future Opportunities and Challenges

Substantial advancements have been achieved in the study of polyimine (PI) and its composites, leading to a deeper understanding of their structure–property relationships and application potentials. However, translating laboratory-scale achievements into large-scale engineering applications remains a major challenge. With the continuous evolution of material systems and the advancement of global carbon neutrality strategies, the development of PI composites faces unprecedented opportunities and technological windows.

First, regarding multifunctional integration, PI materials possess inherent potential such as conductivity, self-healing, flame retardancy, and structural reinforcement. Yet, most current studies focus on optimizing individual properties, lacking comprehensive design strategies tailored for complex service environments. Establishing efficient synergy among dynamic networks, reinforcing phases, and external stimuli-responsive systems demands the development of multiscale collaborative models bridging molecular design, structural architecture, and macroscopic configurations.

Second, limitations associated with practical self-healing and closed-loop recycling hinder widespread adoption. The reconfiguration stability of dynamic bonds under real-world conditions remains insufficient. Developing room temperature reversible bonds (e.g., boronate esters [98], hydrazone bonds [44], and synergistic hydrogen bonding systems [58]) or incorporating low-energy stimuli such as light, humidity, or electrical triggers could significantly broaden their applicability in portable electronics, smart sensors, and medical devices.

Finally, from an industrialization perspective, the lack of standardized, scalable manufacturing platforms restricts the engineering translation of PI composites. Current efforts largely remain at the laboratory scale, with few studies addressing continuous and modular production processes for practical forms such as films, coatings, foams, and sheets. Integrating advanced manufacturing techniques such as 3D printing, intelligent spraying, and roll-to-roll coating could enable the establishment of high-throughput, cost-effective PI material production platforms, accelerating their commercialization.

**Figure 17:**
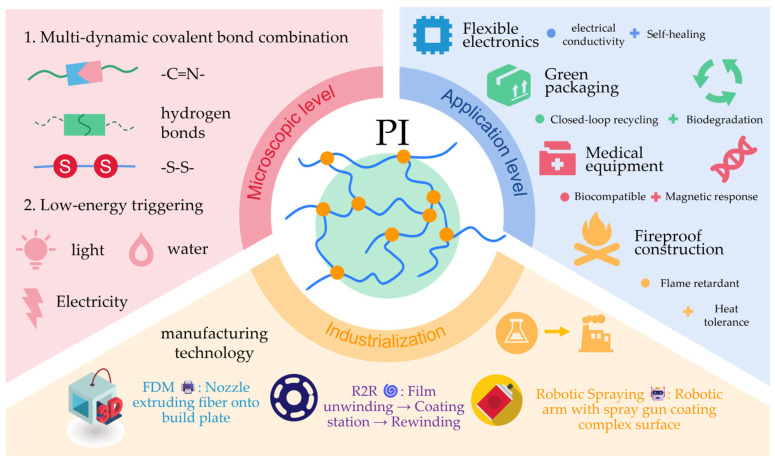
Future outlook schematic: from microscale molecular design to innovations in manufacturing, and ultimately the development trends in the applications of polyimine and its composite materials.

Future developments will require interdisciplinary collaboration, deeply integrating dynamic chemistry, interfacial engineering, sustainable manufacturing, and intelligent responsive systems. Figure 17 provides a forward-looking illustration of the future development landscape of polyimine-based materials. Building a novel, proprietary technology ecosystem for functional materials will be crucial to supporting the advancement toward high-performance, green, and intelligent engineering materials.

## Figures and Tables

**Table 1:** Characteristics of polyimine fabrication methods.

Method	Process Steps	Advantages	Limitations	Applicable Forms	Ref.
Solution Casting	Monomer dissolution → Schiff base reaction → solvent evaporation	Uniform filler dispersion, film formation	High energy consumption, solvent recovery challenges	Films, gels, composites	[20,21]
Hot Pressing	Powder compaction → heating/pressure → molding	Scalability, simplicity	Particle aggregation, weak interfaces	Bulk materials, multilayers	[22,23]
Interfacial Polymerization	Monomer injection → interfacial condensation → film peeling	Ultrathin films, stable interfaces	Harsh reaction conditions, high cost	2D films, functional coatings	[24,25]

**Table 2:** Comparison of representative properties of organic and inorganic reinforcing phases in polyimine composites.

Reinforcement	Type	Key Property Improvements	Primary Characteristics Enhanced	Ref.
Bamboo Powder (BP)	Organic	- Tensile strength: 45.2 MPa (↑17% vs. neat PI) - Young’s modulus: 1.46 GPa (↑194% vs. neat PI)	Toughness and stiffness	[33]
Cellulose Nanofibrils (CNFs)	Organic	- Tensile strength: ↑25% (30 wt% CNF) - Young’s modulus: ↑100% (maximum)	Stiffness	[39]
Carbon Nanotubes (CNTs)	Inorganic	- Electrical conductivity: ↑4 orders of magnitude (10 wt% MWCNT) - Tensile strength: 74 MPa (↑34% vs. neat PI)	Conductivity and strength	[40]
Graphene Nanoplatelets (GnPs)	Inorganic	- Tensile strength: 76.7 MPa (↑7% vs. neat PI) - Flexural strength: 98.2 MPa (↑22% vs. neat PI)	Strength and flexural properties	[41]

“↑” means “increase”.

**Table 4:** Engineering applications of polyimine composites (20 to 25 years).

Abbreviation of Composite	Reinforcement Phase	Composite Method	Key Performance Parameters	Application Field	Specific Engineering Application Cases	Ref.
MBS-TMP	MoS_2_, Te nanowires	Fluid-designed solution shearing	Stable photocurrent (retaining 94.3% after 50,000 bending cycles); high carrier mobility	Flexible electronics	Wearable photodetectors (integrated into textiles for imaging and sensing)	[65]
PI/GP	Graphite–polypropylene	Physical mixing	Self-healing efficiency of 94.6%; output voltage of 1325 V (15 N, 6 Hz)	Energy harvesting	Self-healing triboelectric nanogenerator (TENG) for biomechanical energy collection and wireless transmission	[66]
DIW	Multi-walled carbon nanotubes	Physical mixing + partial curing	Elastic modulus of 520 MPa; tunable conductivity	3D-printed electronic devices	Recyclable 3D-printed sensors (temperature/strain sensors maintaining performance after repair)	[67]
BNNS/PIH TIM	Boron nitride nanosheets	Horizontal centrifugal casting	In-plane thermal conductivity of 7.69 W/m·K; low compressive strength (2.16 MPa)	Electronic thermal management	Heat dissipation materials for 5G base stations and automotive IGBT modules (reducing temperature fluctuation under vibration)	[68]
PETG/CNF-Imine	Polyimine-coated cellulose nanofibers (CNF-Imine)	Melt blending + hot pressing	Tensile strength increased by 12% (with 30 wt% fibers); strong interfacial bonding	Green materials	Industrial manufacturing requiring high strength and stiffness	[39]
PLA/CNF-Imine	Polyimine-coated cellulose nanofibers (CNF-Imine)	Melt blending + hot pressing	Significant stiffness enhancement (up to 30% across fiber range); moderate increases in storage and loss moduli compared to PETG composites	Green materials	Applications requiring stiffness with biodegradable materials	[39]
PI-MWCNT	Multi-walled carbon nanotubes	Chemical linking	97% conductivity recovery; tensile strength of 74 MPa (34% improvement over matrix)	Flexible electronics	Wearable electronic skins (maintaining conductivity after multiple repairs and recycling)	[40]
Polyimine-Metal Complex	Metal ions (Cu^2+^, Mg^2+^, Fe^3+^)	One-pot method	Tensile strength: 2.51 MPa; elongation at break: 1158%; self-healing efficiency: 96.0%; thermal stability (Td5%) > 278 °C	Green materials	Reprocessable and recyclable vitrimers with enhanced thermal, mechanical, solvent, and acid resistance, though poor water resistance	[69]
PI-aGA	Annealed graphene aerogel	In situ polymerization + hot pressing	Tensile strength of 37 MPa; stable conductivity after repeated reshaping	Flexible electronics	High-conductivity composites for flexible circuits and smart wearable devices	[70]
TMPNP	TiO_2_@MXene	Chemical linking	Limiting oxygen index (LOI) of 32%; resistance variation rate of −95% (at 70 °C)	Fireproof materials	Smart building fireproof coatings (cotton fabrics integrated with wireless fire alarms)	[48]
Ionic-PIs	BMIM∙PF_6_	Physical mixing	Tensile strength: 51.8 MPa; elastic modulus: 0.84 GPa; toughness: 1.62 MJ/m^3^; LOI > 27%	Fireproof materials	Material recovery rate exceeding 41.9%; regenerated PI-R membranes retain mechanical strength; enhanced flame retardancy via BMIM∙PF_6_ sacrificial decomposition	[71]
HVP/D230-CF	Carbon fibers	Hot pressing	Tensile strength of 184.4 MPa; LOI of 34.2%	Fireproof materials	Recyclable carbon fiber-reinforced composites for aerospace flame-retardant structures	[72]
PI-NdFeB Soft Robot	Magnetic NdFeB microparticles	Physical mixing + solvent evaporation	Tensile strength of 6.3 MPa; elongation at break of 260%	Biomedical applications	Magnetic soft robots designed for minimally invasive surgical procedures and targeted drug delivery applications	[46]
CF-PI-daMINT	Mechanically interlocked nanotube derivatives (MINTs)	Planetary ball milling + hot pressing	Tensile strength: 68 ± 9 MPa; Young’s modulus: 3.2 ± 0.2 GPa	Green materials	Efficient reinforcement of PI CANs; superior mechanical performance of PI-daMINT composites due to enhanced SWNT dispersion and load transfer	[45]
CFRFP	Carbon fibers	Prepreg compression molding	Tensile stress of 23.7 MPa; water-driven extensibility	Green materials	Weldable/self-healing carbon fiber composites for lightweight automotive parts and recyclable drone structures	[18]
PI-SiCw	Silicon carbide nanowhiskers	Hot pressing	Impact strength improved by 154% (with 2% SiCw); flexural strength of 85.55 MPa	Green materials	High-performance electronic packaging materials (high-temperature and impact resistance)	[73]
GNPs-P	Graphene nanoplatelets	Hot pressing	Tensile strength of 73.05 MPa (at 0.5 wt%); enhanced thermal conductivity	Electronics	LED heat dissipation substrates (replacing traditional metal heat sinks)	[41]
BP/PI	Bamboo powder	Hot pressing	Tensile strength of 45.2 MPa; closed-loop recyclability of 100%	Green materials	Bamboo-based plastic alternatives for eco-friendly packaging and furniture	[33]
RY-PI	Ramie yarn	Chemical linking + interlayer compounding	Tensile strength: 144 MPa; Young’s modulus: 0.97 GPa; elongation at break: 25%	Green materials	High-performance and recyclable natural fiber-reinforced plastic composites (NFRPCs)	[53]
TD	Graphene nanoplatelets (GnPs)	Hot pressing	Tensile strength: 84 MPa; Young’s modulus: 1.6 GPa; thermal conductivity: 1.8 W m^−1^ K^−1^	Electronics	TDG-sn composites for LED chip heat dissipation and thermal management applications	[74]
CFRP	Carbon fibers	Chemical recycling	Tg decreased by 42% in non-woven mat CFRPs; storage modulus (E’) significantly increased; Tg increased by 8% in UD-CFRP	Green materials	Feasibility of recycling fiber-reinforced vitrimer composites, with required process optimizations	[75]
VITRIMER TEDAP	Unidirectional carbon fibers	Hot pressing	Tensile strength: 69 MPa; Tg: 192 °C	Fireproof materials	Enhanced thermal stability and flame retardancy compared to epoxy systems; significant pHRR reduction in vitrimer composites	[76]
PI-FRPs.	Carbon fibers	Powder compression molding	Young’s modulus increased to ~17.3 GPa at 190 °C (1.25 MPa, 4 min)	Repairable coatings	Low-temperature, mold-free in situ repair of polyimine composites, adapted for curved surface applications	[77]
PPCs	Cellulose paper	Hot pressing	Tensile strength: 71 MPa; Young’s modulus: 3.2 GPa	Green materials	Polyimine-filled cellulose papers (PPCs) with excellent mechanical strength, water resistance, gas barrier properties, and recyclability	[43]
WPCs	Wood cellulose	Hot pressing	Transition from brittle to ductile fracture; 13% tensile strength improvement in WPC/25pAPP	Fireproof materials	Improved interfacial interaction and mechanical strength due to imine bond network and mechanical interlocking	[78]

**Table 5:** Comparison of mechanical, thermal, and recycling properties of high-performance thermosetting polymers.

Polymer	Mechanical Properties	Thermal Properties		Recyclability
	Tensile Strength	Tg	Td5%	Recyclable
Epoxy	55–85 MPa	105–120 °C	250–350 °C	×
Phenolic Resin	30–60 MPa	100–150 °C	260–350 °C	×
Bismaleimide (BMI)	60–120 MPa	230–350 °C	400–500 °C	×
Polyimine (PI)	30–96 MPa	−20–100 °C	250–400 °C	√

**Table 6:** Comparison of pHRR parameters between polyimine vitrimer and PER epoxy resin matrix (3% phosphorus content) [76].

Parameter	Per Tedap	Vitrimer App	Vitrimer Rdp	Vitrimer Tedap
pHRR (kW/m^2^)	111	175	290	218
Time to pHRR (s)	110	234	207	260
Parameter	Per Composite	Vitrimer Composite	Vitrimer App Composite	Vitrimer Rdp Composite
pHRR (kW/m^2^)	351	289	186	152
Time to pHRR (s)	39	140	158	176

## Abbreviations

The following abbreviations are used in this manuscript:

PI	Polyimine
DCC	Dynamic Covalent Chemistry
CANs	Covalent Adaptive Networks
SWNTs	Single-Walled Carbon Nanotubes
MWCNT	Multi-Walled Carbon Nanotube
CNTs	Carbon Nanotubes
COF	Covalent Organic Framework
CF	Carbon Fiber
TENG	Triboelectric Nanogenerator
TEGs	Thermoelectric Generators
SSEs	Solid-State Electrolytes
TFC	Thin-Film Composite
MINTs	Mechanically Interlocked Nanotube Derivatives
BP/PI	Bamboo Powder/Polyimine Composite
PVP	Polyvinylpyrrolidone
TREN	Tris(2-aminoethyl)amine
TPA	Terephthalaldehyde
PGCS	Peach Gum Polysaccharide/Chitosan Composite
IPIN	Ionic Polyimine Network
ASA	Aerogel-Sol-Aerogel Process
TMPNP	TiO_2_@MXene/P, N-containing Polyimine Nanocomposite
CFRPs	Carbon Fiber-Reinforced Composites
RY-PI	Ramie Yarn-Reinforced Polyimine Vitrimer Composites
PPCs	Paper–Polyimine Composites
BNNS/PIH TIM	Boron Nitride Nanosheets/Polyimine Hybrid Thermal Interface Material
SEM	Scanning Electron Microscopy
TEM	Transmission Electron Microscopy
FTIR	Fourier Transform Infrared Spectroscopy
TGA	Thermogravimetric Analysis
DTG	Derivative Thermogravimetric
ECL	Electrochemiluminescence
Vitrimer	A polymer combining thermoset properties with dynamic covalent adaptability
